# The Effect of Hospital Boarding on Emergency Medicine Residency Productivity

**DOI:** 10.5811/westjem.31064

**Published:** 2024-11-27

**Authors:** Peter Moffett, Al Best, Nathan Lewis, Stephen Miller, Grace Hickam, Hannah Kissel-Smith, Laura Barrera, Scott Huang, Joel Moll

**Affiliations:** Department of Emergency Medicine, Virginia Commonwealth University School of Medicine, Richmond, Virginia

## Abstract

**Introduction:**

Emergency department boarding has escalated to a crisis, impacting patient care, hospital finances, and physician burnout, and contributing to error. No prior studies have examined the effects of boarding hours on resident productivity. If boarding reduces productivity, it may have negative educational impacts. We investigated the effect of boarding on resident productivity as measured by patients per hour and hypothesized that increased boarding leads to decreased productivity.

**Methods:**

This was a retrospective study at a quaternary, urban, academic Level I trauma center from 2017–2021 with a three-year emergency medicine residency of 10–12 residents per year and annual volumes of 80,000–101,000. Boarding was defined as the time between an admission order and the patient leaving the ED. We created a multivariable mixed model with fixed covariates for year, month, day of week, resident experience, shift duration, total daily ED patients, and with residents as repeated measures. The effect of boarding was estimated after covarying out all other factors.

**Results:**

All variables included in the model were significantly associated with changes in productivity. Resident experience has the largest effect such that for each month of residency experience, a resident adds 0.012 patients per hour (95% confidence interval [CI] 0.010–0.014). Isolating the effect of boarding demonstrated that for every additional 100 hours of boarding, a resident’s productivity decreased by 0.022 patients per hour (95% CI 0.016–0.028). In the study, the median daily boarding was 261 hours; if this were eliminated (assuming a resident completes 100 10-hour shifts annually), a resident could be expected to see 56.9 more patients per year (95% CI 40.7–73.1).

**Conclusion:**

Hospital boarding significantly reduces resident productivity as measured by patients per hour. Further studies are warranted to determine the educational impact.

Population Health Research Capsule What do we already know about this issue?

*Emergency department boarding negatively impacts patient care, hospital efficiency, and physician well-being.*

What was the research question?

*Does increased ED boarding reduce emergency medicine resident productivity, as measured by patients per hour?*

What was the major finding of the study?

*For every additional 100 hours of ED boarding, a resident’s productivity decreased by 0.022 patients per hour (95% CI 0.016–0.028); a resident sees 57 fewer patients per year due to boarding.*

How does this improve population health?

*Understanding the negative effects of boarding on productivity may help policy makers find solutions to improve patient flow, patient care, resident education, and overall health outcomes.*

## INTRODUCTION

Emergency department (ED) boarding (defined as patients admitted to the hospital but remaining in the ED) has reached critical levels and has been declared a crisis by the American College of Emergency Physicians.^1^ The scope of the crisis is daunting with effects on patient care, errors, physician burnout, hospital economic stress, and ambulance diversion.^2^ Increased ED boarding also leads to increases in medication errors, time to antibiotics, time to percutaneous coronary intervention for patients with myocardial infarction, time to care for patients with acute stroke, patient mortality, and risk-adjusted hospital spending, and has effects on all levels of acuity.^3^ ^–^ ^10^

Within the context of boarding, EDs must also provide sound educational training involving both quality and quantity of patient experiences. Residency programs seek to improve efficiency and productivity in their residents throughout their training. Many variables have been associated with resident productivity including time of shift, shift length, and resident experience.^11^ ^–^ ^13^ There are, however, few studies that evaluate the effect of ED crowding and boarding time on the effect of emergency medicine (EM) resident productivity.^14^ If boarding decreases the number of patients seen during a residency, there may be an impact on resident education.

In this study we aimed to investigate the effect of boarding on EM resident productivity as measured by patients per hour. We hypothesized that increased hospital boarding would result in decreased resident productivity.

## METHODS

### Study Design and Setting

This was a retrospective study conducted at the Virginia Commonwealth University Health System, the only comprehensive Level I trauma center in Richmond, VA. During the study period from January 2017–June 2021, the total patient volumes ranged from 80,000–101,000 per year. On average, 30% of patients were admitted to the hospital, of whom 5% went to the intensive care unit. Patients <18 years of age constituted 22% of the total volume. The department is staffed with board-certified emergency physicians, and during the study period 81% of patients were seen by a resident. The remaining non-resident cases were seen by advanced practice practitioners (APP) in a low-acuity area of the ED or by attending physicians and were not included in the study. Throughout the study there was no change in this staffing model such that APPs were never competing for the same patients as residents. The department has 76 beds with 35 in an acute area, 10 in trauma/resuscitation, 10 in a mid-track area, 16 in a pediatric department, and five in a fast-track zone.

Our residency program is three years in length, and class sizes ranged from 10 residents in 2017 to 12 residents in 2021. During postgraduate years (PGY)-1, 2, and 3, residents work in the ED for 26 weeks, 29 weeks, and 35 weeks, respectively. Resident shift lengths varied from 9–12 hours with the most typical shift being 10 hours. On average, each 24-hour period had a total of 137 hours of resident coverage in overlapping shifts. The EM residents saw patients in all Emergency Severity Index (ESI) categories and were the primary physicians for all emergent patients (ESI 1 and 2). Residents cared for patients in all areas of the ED other than the low-acuity area. All residents staff patients directly with an attending physician without oversight by a more senior resident; therefore, the productivity numbers for residents in all three years of training are independent.

The study was granted exempt status by the Virginia Commonwealth University Institutional Review Board (HM20024717).

### Selection of Participants

Data from all patients evaluated by an EM resident was captured in a database, and in conjunction with scheduling data it was used to determine the average number of patients per hour. Only EM residents were included. The study period was selected as this was the maximum amount of time for which data was available prior to the hospital switching to a new electronic health record. As the database was initially created to provide feedback to residents, certain data was removed and not available to us for analysis. Information from the first month of EM for each resident was not provided, and due to initial effects from the COVID-19 pandemic, data from April–July 2020 was not included.

### Measurements

We combined three databases for analysis: the patient database of all ED encounters; the resident scheduling database; and the hospital boarding database.

During the study period, the EM residency program received monthly, system-generated reports listing the unique patient identifier, name of the resident assigned to care for the patient, the ESI acuity level, the date/time of first contact and check out, and the disposition. The resident assignment was derived from tracking board data, and in scenarios where multiple residents were assigned to a patient encounter, only the first resident assigned was credited for each unique patient encounter. The EM residents were scheduled for 9-hour, 10-hour, or 12-hour shifts during the study period. All non-EM residents and staff were excluded from the patient database.

Boarding data was reported daily from hospital analytics. The number of hours of boarding was defined as the time between an admission order and when the patient left the ED. Boarding hours was selected as this was the variable available to us from the hospital analytics database.

### Outcomes

We designed a model to isolate the effects of ED boarding on resident productivity as measured by patients per hour. Patients per hour was defined as the total number of new patients seen during the shift divided by the duration of the shift in hours. The covariates were chosen from those found in previous studies to be related to resident productivity.^11^ ^,^ ^13^ ^,^ ^15^ ^,^ ^16^ These included year, month, day of the week, cumulative residency months in training, shift duration, total patients per day, and boarding. Months in training was chosen as a continuous covariate to delineate resident experience rather than the rough classification of PGY-1, -2, or -3 based on the observation that resident productivity begins low in the PGY-1 year, increases in the PGY-2 year, and then plateaus. This monthly experience variable was modeled using cubic regression.

### Analysis

We described the data using counts and percentages. Patients per hour was modeled using a multivariable mixed model, with covariates defined as fixed effects and residents as repeated measures. We used an autoregressive (AR1) covariance structure to account for the dependence between repeated measures. The fixed effects were year (reference = 2019), month (reference = 12), day of the week (reference = Thursday), resident month in training (centered on 18), total patients per day/100, shift duration, and daily boarding hours/100. We chose the year 2019 as a reference as it was the last full year of data prior to the start of the COVID-19 pandemic. December was chosen as it aligns with the 18^th^ month of residency, which is when productivity plateaued in our model. Thursday was selected as it is thought to represent the day with the most ideal flow since it avoids weekends, Monday, and Friday patient surges, as well as Wednesday morning didactics when EM residents are not working clinically. The total patients per day, shift duration, and boarding hours were referenced at the median values in our dataset.

We estimated the effect of boarding from the marginal regression model after covarying out all other factors. Estimates are described using 95% confidence intervals. All data management and analysis were performed using SAS software (version 9.4 and JMP Pro version 17.2 (SAS Institute Inc, Cary, NC).

## RESULTS

### Characteristics of Study Subjects

During the study period, 263,058 patients were seen in the ED by 601 clinicians including the 80 EM residents studied. During the 49 months studied between 2017–2021, EM residents were scheduled to 16,949 shifts and were assigned 188,685 patients (Table 1). Total daily patient volume varied considerably during this time (mean 177, SD 26, range 88–263). As indicated in the table, the ED experienced a patient count variability that changed across years, months, days of the week, shifts, and PGY level. Of all 188,167 patients seen by EM residents, 40% were admitted.

**Table 1.** Characteristics of the emergency department residents’ shifts and patients evaluated (January 2017–June 2021).

Characteristic	Shifts N	Patients N	(%)
Total	16,949	188,685	
Year			
2017	3,496	44,119	(23)
2018	3,955	47,569	(25)
2019 (11 months)*	4,053	47,035	(25)
2020 (8 months)†	3,101	29,191	(15)
2021 (6 months)	2,344	20,771	(11)
Month			
1- January	1,909	21,052	(11)
2- February	1,576	18,004	(10)
3- March	1,680	18,901	(10)
4†- April	1,302	15,229	(8)
5†- May	1,371	16,385	(9)
6†- June	1,337	15,191	(8)
7†- July	820	10,129	(5)
8- August	1,560	15,543	(8)
9- September	1,376	14,741	(8)
10- October	1,431	15,299	(8)
11*- November	1,062	11,639	(6)
12- December	1,525	16,572	(9)
Day of week			
Sunday	2,249	25,887	(14)
Monday	2,679	29,099	(15)
Tuesday	2,756	29,504	(16)
Wednesday^‡^	1,989	21,970	(12)
Thursday	2,601	27,874	(15)
Friday	2,525	28,785	(15)
Saturday	2,150	25,566	(14)
Shift			
7 am to 5 pm	1,688	16,332	(9)
7 am to 7 pm	180	2,512	(1)
9 am to 7 pm	2,546	28,306	(15)
12 pm to 10 pm	3,386	38,586	(20)
2 pm to 12 am	2,470	28,631	(15)
3 pm to 12 am	3,553	41,138	(22)
9 pm to 7 am	3,126	33,180	(18)
PGY			
PGY-1^§^	5,162	44,817	(24)
PGY-2	4,756	57,447	(30)
PGY-3	7,031	86,421	(46)
Disposition			
Admitted		74,663	(40)
Discharged		114,022	(60)

* November 2019 was excluded as the hospital information management system was down.

† April 2020 through July 2020 was excluded due to COVID-19 and hospital changes.

‡ Wednesdays mornings are resident didactics.

§ The first month of a residency was excluded (orientation month).

*ESI*, Emergency Severity Index; *PGY*, postgraduate year.

**Figure 1.**
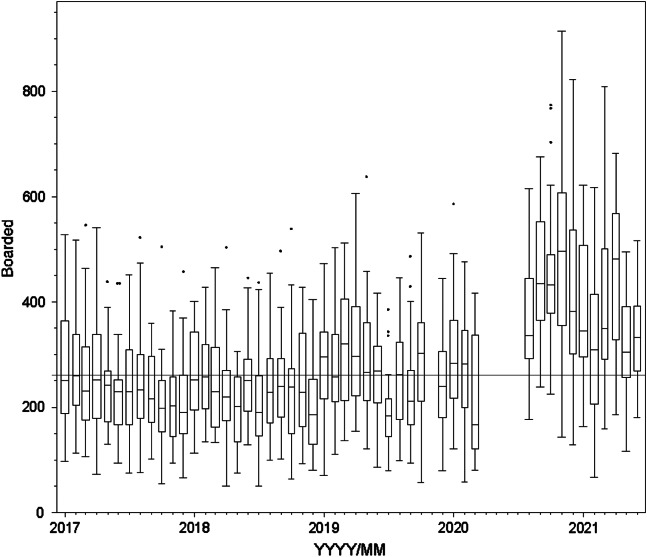
Boarding across study years. Line set at median boarding hours across the entire study period (261 hours/day). Each box plot represents a month (line = median, box = 25^th^ to 75^th^ quartile, whiskers = typical extremes, circles = outliers). Note: April 2020–July 2020 hours are not available as they correspond to the beginning of the COVID-19 pandemic.

Boarding hours per day varied considerably (mean 281, SD 127, range 50.8–914.4; Figure 1). The hospital information system calculated boarding hours daily; however, across the 1,490 days studied, there were six impossible (negative) values and nine very low values. Low values were identified by large residuals in the multiple regression model. Rather than treating these as missing values, we used a multiple regression model to impute the 15 values in question.

### Main Results

**Figure 2.**
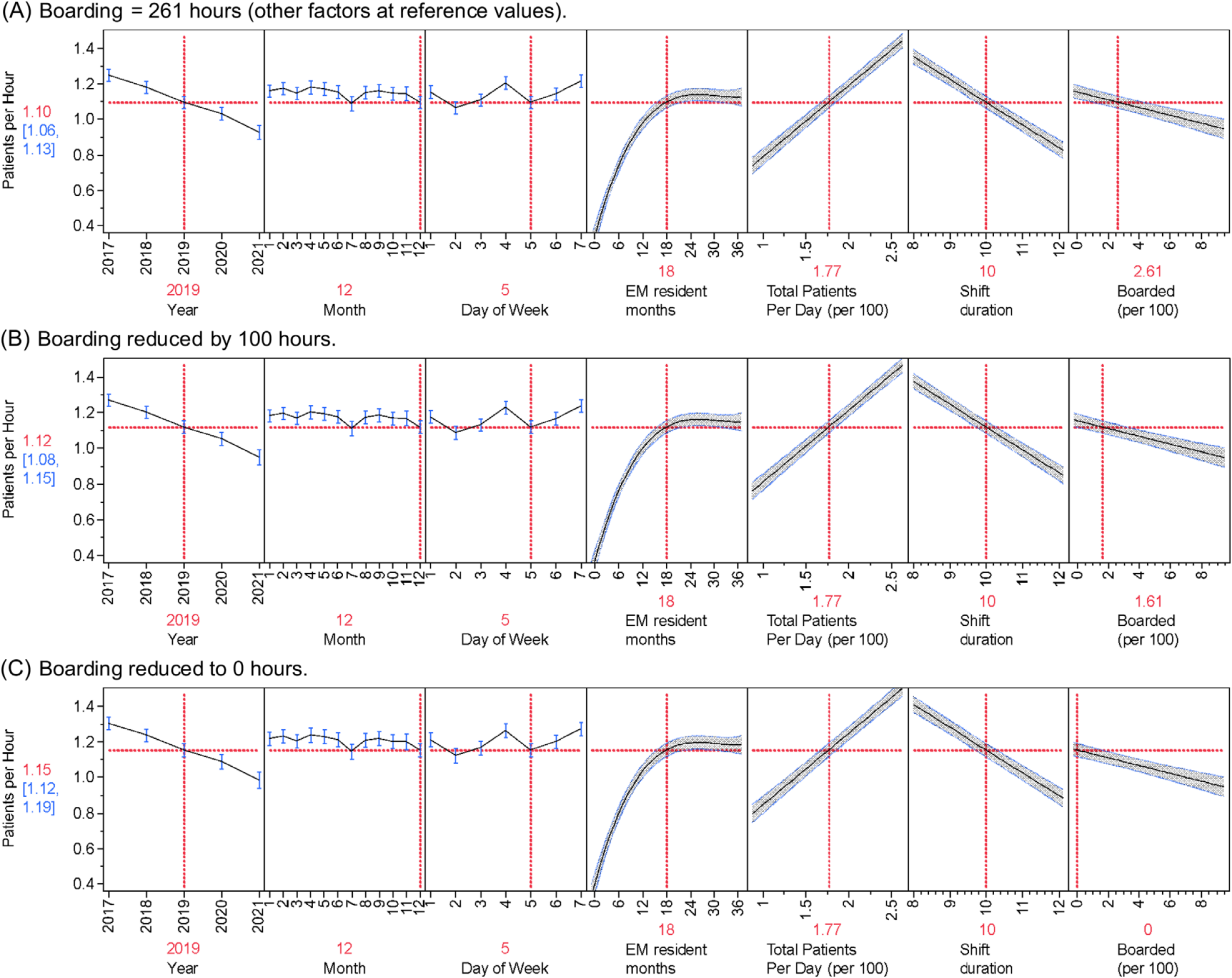
Multiple regression results predicting new patients per hour per resident for each variable. All values (year, month, day of week, EM resident months, total patients, shift duration) in model held at reference standards with adjustments to boarding (last panel of each graph). Expected patients per hour in each scenario is indicated by the red number in the Y axis with 95% confidence intervals in blue. As boarded hours change (last panel of each graph) so do patients per hour (red number to left of each graph) in each of the three scenarios (A: Median boarding of 261 hours. B: Reducing boarding by 100 hours. C: Eliminating boarding hours.)

All the factors in the repeated-measures mixed-model were significant (*P* < 0.001). Table 2 shows the estimated effect of each term in the model. The joint effect of all the factors on resident productivity is shown in Figure 2. These profile plots show the marginal model predicted value of resident productivity on the vertical axis across all the covariates on the separate horizontal axes. The importance of a factor is visualized by the steepness of the prediction trace.

**Table 2.** Multiple regression results predicting new patients per hour per resident for each variable.

Effect	Estimated new patients per hour	Standard error	95% CI
Intercept	1.0957	0.0173	1.0618 to 1.1297
Year				
2017	0.1501	0.0122	0.1262 to 0.1740
2018	0.0837	0.0117	0.0608 to 0.1065
2019	[reference]			
2020	−0.0641	0.0137	−0.0909 to −0.0373
2021	−0.1682	0.0156	−0.1987 to −0.1377
Month				
1- January	0.0635	0.0172	0.0298 to 0.0972
2- February	0.0776	0.0182	0.0420 to 0.1133
3- March	0.0498	0.0181	0.0144 to 0.0852
4- April	0.0840	0.0197	0.0453 to 0.1227
5- May	0.0750	0.0196	0.0366 to 0.1133
6- June	0.0585	0.0201	0.0191 to 0.0979
7- July	−0.0077	0.0219	−0.0507 to 0.0353
8- August	0.0550	0.0185	0.0188 to 0.0912
9- September	0.0654	0.0187	0.0288 to 0.1021
10- October	0.0487	0.0184	0.0127 to 0.0847
11- November	0.0486	0.0199	0.0095 to 0.0876
12- December	[reference]			
Day of the week				
Sunday	0.0587	0.0118	0.0357 to 0.0818
Monday	−0.0312	0.0118	−0.0542 to −0.0082
Tuesday	0.0122	0.0110	0.0094 to 0.0338
Wednesday	0.1094	0.0123	0.0854 to 0.1334
Thursday	[reference]			
Friday	0.0475	0.0109	0.0261 to 0.0688
Saturday	0.1182	0.0120	0.0948 to 0.1417
Resident months (linear)*	0.0122	0.0010	0.0101 to 0.0142
(quadratic)	−0.0011	0.0000	−0.0012 to −0.0010
(cubic)	0.00003	0.00001	0.00002 to 0.00004
Total patients per day (per 100 patients)*	0.4021	0.0165	0.3697 to 0.4344
Shift duration*	−0.1277	0.0070	−0.1413 to −0.1140
Boarded (per 100 hours)*	−0.0218	0.0032	−0.0280 to −0.0156

The mixed-model also included resident as a repeated-effect with an AR(1) covariance structure.

* Continuous covariates were referenced to the median value. Median resident month = 18, total patients per day/100 = 1.77, shift duration = 10 hours, boarded hours/100 = 2.61.

*CI*, confidence interval.

Isolating the effect of boarding demonstrated that for every additional 100 hours of daily departmental boarding, individual resident productivity decreased by 0.022 patients per hour (95% confidence interval [CI] 0.016–0.028, Table 2). In the reference standard scenario, a resident could be expected to see 1.10 patients per hour with boarding at the daily median (261 hours) but could see 1.15 patients per hour if boarding were eliminated (Figure 2, Panel C). Table 3 shows how resident productivity was degraded by boarding across the range of values seen at our institution. A resident would see 1.14 patients per hour when boarding was at the lowest in the study compared to 0.95 patients per hour at the maximum level of boarding seen in the study, which is a difference of 0.19 patients per hour (95% CI 0.15–0.22). Assuming a resident completes approximately 100 shifts a year that are of 10 hours duration and boarding was eliminated, then a resident could be expected to see 56.9 more patients per year (95% CI 40.7–73.1). This would represent a 5% increase in patient volume per resident annually.

**Table 3.** Estimated resident productivity by boarding hours.

Cutoff	Boarded (hours)	Estimated patients per hour	Standard error	95% CI
Maximum	914	0.954	0.027	0.900 to 1.007
75th percentile	351	1.076	0.018	1.042 to 1.111
Median	261	1.096	0.017	1.062 to 1.130
25th percentile	189	1.111	0.017	1.077 to 1.146
Minimum	51	1.141	0.018	1.105 to 1.178
No boarding	0	1.153	0.019	1.115 to 1.190

Marginal estimates from the mixed model with the following factors held constant: year = 2019, month = 12, day of the week = 5 (Thursday), resident month in training = 18, total patients per day/100 = 1.77, shift duration = 10 hours.

*CI*, confidence interval.

Resident experience has the largest effect on resident productivity. Resident productivity was low initially at 0.5 patients per hour (95% CI 0.46–0.54) by the second month of training (Figure 2). Improvement was initially rapid to 0.75 patients per hour at seven months, then plateaued near the 18-month point (1.10 patients per hour) to finally reach 1.12 patients per hour at the end of the 36 months (95% CI 1.08–1.17). When evaluating our data by PGY level, our PGY-1 residents saw 0.75 per hour, PGY-2 residents saw 1.10 patients per hour, and PGY-3 residents saw 1.12 patients per hour.

Total patients per day presenting to the ED was the next most important factor in resident productivity. For every 100 new patients presenting to the ED, an individual resident would be expected to add 0.40 patients per hour (95% CI 0.37–0.43). The median value for daily total patient volume was 177 patients per day, but a low-volume day at the 10^th^ percentile (143 total patients) resulted in a corresponding decrease in resident productivity to 0.96 patients per hour (95% CI 0.92–1.00). For a high-volume day at the 90^th^ percentile (210 patients), resident productivity increased to 1.23 patients per hour (95% CI 1.19–1.26).

Resident productivity also changed based on the year, shift duration, and day of the week. Resident productivity was highest in 2017 at 1.25 patients per hour (95% CI 1.21–1.28) and steadily decreased to the 0.93 patients per hour seen in 2021. Resident productivity for a nine-hour shift was predicted to be 1.21 patients per hour (95% CI 1.19–1.26), whereas for a 12-hour shift it was predicted to be 0.84 patients per hour (95% CI 0.80–0.89). Saturdays and Wednesdays averaged approximately 1.21 patients per hour, Sundays, and Fridays approximately 1.15 per hour, and Mondays, Tuesdays, and Thursdays 1.10 patients per hour.

Month-to-month variability had the smallest effect on resident productivity. Compared with the other months, July and December had lower resident productivity (1.09 vs 1.16 patients per hour).

## DISCUSSION

To our knowledge, this is the first study to demonstrate that there is a significant reduction in resident productivity (measured as patients per hour) due to hospital boarding in the ED. In our model, this resulted in a decrease of 0.022 patients per hour (95% CI 0.016–0.028) for every 100 hours of daily boarding. While performed at a single institution, our dataset broadly aligns with multiple studies previously completed regarding resident productivity. In our study, we analyzed resident experience as the number of months in training rather than divided into PGY level. This was based on our observation that productivity rapidly increased during the PGY-1 year and then plateaued in the middle of the PGY-2 year.

When evaluating our data by PGY level, our PGY-1 residents saw 0.75 patients per hour, PGY-2 residents saw 1.10 patients per hour, and PGY-3 residents saw 1.12 patients per hour. Prior studies have demonstrated similar patterns with PGY-1 to -3 residents seeing between 0.79–0.81 patients per hour, 1.05–1.2 patients per hour, and 1.22–1.27 patients per hour, respectively.^17^ ^–^ ^19^ A study by Henning et al showed rapid progression from PGY-1 to PGY-2 year and then gradual progression in PGY-3 year but was based on patients per day.^20^ Similarly, a study by Turner-Lawrence and Todd saw increasing productivity from 1.2 patients per hour to 1.5 patients per hour to 1.6 patients per hour by PGY-1 to -3 residents, respectively.^13^ While these productivity numbers are higher than those in our study, the authors did not adjust for additional variables.

In a more comparable study, Kirby et al reported the efficiency of EM residents during ED crowding.^14^ The authors used the National Emergency Department Overcrowding Study (NEDOCS) scoring system to categorize states in the ED as not crowded, crowded, and overcrowded. They found that resident productivity measured as new patients per hour increased initially in all year groups as the ED transitioned from not crowded to crowded, but then remained stable when transitioning from crowded to overcrowded. While the NEDOCS score uses a measure of ED boarding (the waiting time of the longest admitted patient), it does not include total patient boarding hours as in our study. Our study more directly examines the effect of boarding (one element of crowding) on resident productivity. The paradoxical increase in resident productivity in the Kirby study may have been due to an increased number of patients presenting to the ED, which could have increased the NEDOCS score. Our study demonstrated that resident productivity increased with higher patient volumes, and including this in our model allowed us to better isolate the effect of boarding.

According to a study by the Academy of Administrators in Academic Emergency Medicine and the Association of Academic Chairs of Emergency annual benchmark survey, boarding times have dramatically increased since the COVID-19 pandemic.^21^ By the end of their study period, the median number of boarding hours per month was 11,480, which approximates to 382 hours of daily boarding. In our study, which includes a pre-pandemic period, the median daily boarding was 261 hours, suggesting that boarding is likely worsening over time and is a problem at many academic medical centers.

The educational impact of decreased patient volumes caused by boarding is uncertain. It is reasonable to expect that residents seeing fewer cases may lose valuable learning opportunities, but this has not been well studied and no firm numbers exist to suggest a threshold at which education suffers. Prior authors have surveyed residents regarding a perceived decrease in education during crowding.^22^ ^,^ ^23^ These studies concluded that residents did not perceive a difference in education during these times, but they used differing measures of crowding, were survey-based, and underpowered. Educators may switch to different models of teaching during periods of high boarding, leading to residents perceiving a less deleterious effect.^24^

Others have postulated an educational Starling effect whereby some boarding allows supervising physicians more time to teach, but at some point there are diminished returns as fewer new patients become available to discuss.^25^ A more recent study was conducted during the current boarding epidemic; the authors surveyed EM program directors regarding their perceptions of the impact of boarding on resident training.^26^ In this study, 80% of the respondents felt that boarding negatively affected resident education, especially in the domains of managing department throughput and managing high volumes of patients per resident. While survey-based in nature, the study results broadly aligns with the prior studies in this area.

Theoretically, residents who see fewer cases may lose valuable learning opportunities. While the components of Bloom’s domains of educational activities can be learned via different modalities of instructional techniques, clinical experience allows for the linking of knowledge to skills and then to attitudes/emotions.^27^ By decreasing a learner’s exposure to patients, one could argue that residents may lose valuable experiential learning opportunities. While some of these can be replicated in simulation or case-based discussion, other skills cannot and are best learned via hands-on, experiential learning encounters. Experiential learning theory, as described by Kolb, highlights the importance of real-life experience and the influence this has on learning.^28^ Unlike traditional learning and instructional methodology that focuses on rote memorization, experiential learning is an active process where residents are engaged in concept transformation through action as well as reflection on their experiences and patient encounters.

This learning theory also emphasizes principles of adult education in which prior learning experiences can be leveraged to create more meaningful and relevant educational experiences.^29^ Additionally, decreasing patient interaction may also affect residents’ application and translation of knowledge into practice. Behavioral learning theory emphasizes learning through interactions with the environment where reinforcement and feedback can encourage modification of behaviors. By incorporating behavioral learning strategies, medical education can foster not only technical competencies but also the development of professional habits such as effective communication between team members and patients.^30^

## LIMITATIONS

This study has several limitations. This was a single-center study that took place in a high acuity, quaternary-care center that also experiences high levels of boarding, which may limit generalizability to other centers. The database that captured the resident patient assignment was based on tracking board data and may have occasionally miscredited a resident with a patient encounter; however, as the dataset was large and involved multiple years with complete datasets for three full classes of residents this is unlikely to have greatly influenced the data. Our resident class size did increase during the 2021 year and thus could theoretically have decreased the number of patients available per resident. While we did not study that directly, it is unlikely to have impacted the data greatly as the additional residents allowed for the creation of an outside rotation at a free-standing emergency center and, therefore, resident staffing hours stayed generally consistent at the study site.

Our model did not include a measure of patient acuity as a covariate. While the ESI category and disposition were recorded for each patient, we did not feel there was a reliable way to convert this data into a meaningful measure of hourly acuity that influenced the amount of time a resident might dedicate toward patient care. For example, an ESI-1 patient who is admitted for an ST-segment elevation myocardial infarction may stay in the department for 15 minutes leaving the bed open for a new patient, while an ESI-3 patient requiring a workup for abdominal pain including imaging who is discharged may occupy a room and a resident for multiple hours. Since our dataset was large, it was assumed that all residents would be exposed equally to the same mix of acuities on individual shifts, by the end of their residency and thus limit the effect on the data. Additionally, recent studies have called into question the accuracy of the ESI.^26^ ^,^ ^27^ A prior study on resident productivity did not show a correlation between ESI and clinician disposition times.^14^

Our study also included data from the COVID-19 pandemic, which affected patient volumes and ED boarding. The dataset we used was initially meant for reporting individual residents’ productivity measures, so data from the first few months of the pandemic was not available for our current study. This likely served to decrease the effect of the initial pandemic response on our data. Just prior to the pandemic our ED had seen a growth in patient volumes from 87,000 patients per year to a peak of 101,000 patients per year, which was followed by a rapid decline to 83,000 a year in the 2021–2022 year. The volumes did slowly rise after the study period. This may have influenced some of the data from our later resident-year groups and served to decrease productivity.

Our measure of boarding may also have limitations. Total boarding hours per day was the variable available from our hospital analytics department. The number of boarded patients per day may have provided different data. For example, in our model a single behavioral health patient boarding for 20 hours from one day would be indistinguishable from 20 patients boarding in 20 individual rooms for a single hour each. As the dataset is large, and all residents were exposed to the same conditions throughout their time, it is unlikely any one resident’s data (or the trend) would be affected based on these types of outliers.

## CONCLUSION

We found a significant reduction in resident productivity as measured by patients per hour during periods of increased boarding. Further studies are warranted to determine the educational impact of these findings.
